# Membrane Kymograph Generator: a cross-platform GUI software for automated generation and analysis of kymographs along dynamic cell boundaries

**DOI:** 10.1093/bioinformatics/btag400

**Published:** 2026-06-18

**Authors:** Tatsat Banerjee, Bedri Abubaker-Sharif, Peter N Devreotes, Pablo A Iglesias

**Affiliations:** Department of Cell Biology and Center for Cell Dynamics, School of Medicine, Johns Hopkins University, Baltimore, MD 21205, United States; Department of Chemical and Biomolecular Engineering, Whiting School of Engineering, Johns Hopkins University, Baltimore, MD 21218, United States; Department of Cell Biology and Center for Cell Dynamics, School of Medicine, Johns Hopkins University, Baltimore, MD 21205, United States; Department of Biomedical Engineering, Whiting School of Engineering and School of Medicine, Johns Hopkins University, Baltimore, MD 21205, United States; Department of Cell Biology and Center for Cell Dynamics, School of Medicine, Johns Hopkins University, Baltimore, MD 21205, United States; Department of Biological Chemistry, School of Medicine, Johns Hopkins University, Baltimore, MD 21205, United States; Department of Cell Biology and Center for Cell Dynamics, School of Medicine, Johns Hopkins University, Baltimore, MD 21205, United States; Department of Biomedical Engineering, Whiting School of Engineering and School of Medicine, Johns Hopkins University, Baltimore, MD 21205, United States; Department of Electrical and Computer Engineering, Whiting School of Engineering, Johns Hopkins University, Baltimore, MD 21218, United States

## Abstract

**Summary:**

The plasma membrane and accompanying cortex serve as major hubs of signal transduction and cytoskeletal activities that collectively regulate cell physiological processes such as migration, polarity, macropinocytosis, phagocytosis, and cytokinesis. Yet, dynamically tracking membrane-cortex associated protein or lipid kinetics from live-cell image series remains challenging, primarily due to the difficulty of accurately extracting and aligning the cell boundary between consecutive frames as the cell continuously deforms and moves. Here, we present *Membrane Kymograph Generator*, a cross-platform software that accepts multichannel time-lapse live-cell fluorescent imaging datasets and automates boundary tracking, inter-frame alignment, and intensity sampling along the boundary. The software implements a rotational offset minimization algorithm that aligns boundaries across consecutive frames by exhaustively searching for the optimal angular shift that minimizes point-to-point distances, while handling variations in boundary point counts due to cell shape changes. The software outputs kymographs representing spatiotemporal dynamics of membrane-associated proteins or biosensors, allows users to fine-tune visualization parameters through an interactive interface, and provides built-in correlation analysis tools for multi-channel datasets. Furthermore, a native Python API enables programmatic usage for batch processing and further downstream analysis. Validation tests demonstrated that the *Membrane Kymograph Generator* accurately tracks, visualizes, and quantitates the spatial kinetics of a wide array of membrane proteins and lipid biosensors over extended time periods, in a variety of cell types including *Dictyostelium* amoeba, human neutrophils, mouse macrophages, and mammalian cancer cells. The GUI-based software is user-friendly, requires no technical expertise, and significantly reduces the manual effort required for kymograph generation and analysis while ensuring high accuracy and reproducibility.

**Availability and Implementation:**

Membrane Kymograph Generator is free and open-source, licensed under GNU General Public License 3.0 or later. It can be installed on both x86-64 and AArch64/ARM64 computers running Windows, macOS, or any standard Linux distribution. The software is distributed as standalone installer files and portable executables targeting specific architectures and operating systems, requiring no dependency resolution. The source code, documentation/wiki, installers, portable binaries, and test data are freely available at https://github.com/tatsatb/membrane-kymograph-generator. The software can also be installed via PIP (package ID: *membrane-kymograph*, https://pypi.org/project/membrane-kymograph) and accessed programmatically via a built-in Python API. The source code is also archived on Zenodo (DOI: 10.5281/zenodo.20318834).

## 1 Introduction

The plasma membrane and associated cortex undergo rapid remodeling driven by coordinated activities of lipid and protein components that collectively define signal transduction and cytoskeletal organizations, and thereby regulate cell physiological processes such as migration, polarity, macropinocytosis, phagocytosis, and cytokinesis (Ridley *et al.* 2003, Swaney *et al.* 2010, Banerjee *et al.* 2025). Advances in live-cell fluorescence microscopy and genetically-encoded biosensors have enabled monitoring of membrane dynamics with high spatiotemporal resolution, yet quantitative analysis remains challenging due to the curved, complex geometry of cell membranes and the difficulty of decoupling morphological changes from protein or lipid dynamics.

Kymographs, two-dimensional space-time plots representing spatial position and fluorescence intensity over time, have emerged as powerful visualization tools in cell biology and biophysics. However, generating accurate membrane kymographs from time-lapse datasets remains non-trivial, as cell boundaries undergo large-scale deformations during dynamic processes. While deep learning-based tools such as ilastik (Berg *et al.* 2019), CellPose (Stringer *et al.* 2021), CellTracker (Hu *et al.* 2021), Celldetective (Torro *et al.* 2025), DeepCell (Greenwald *et al.* 2022), and CellProfiler (Kamentsky *et al.* 2011, Stirling *et al.* 2021) can accurately segment cell boundaries, they do not track and align boundaries that assume different sizes and geometries between frames. Existing Fiji/ImageJ (Schindelin *et al.* 2012) kymograph plugins such as *KymographBuilder* (Mary *et al.* 2016) and *Multi Kymograph* (https://github.com/fiji/Multi_Kymograph) (Rietdorf and Seitz 2017) only work with static, user-defined lines. Consequently, researchers have relied on manual or semi-automated approaches, analyzed straight-line membrane segments, or developed custom code: methods that are time-consuming, prone to user-bias, and often inaccessible to experimental biologists (Bosgraaf *et al.* 2009, Arai *et al.* 2010, Bosgraaf and Van Haastert 2010, Tyson *et al.* 2010, Xiong and Iglesias 2010, Barry *et al.* 2015, Miao *et al.* 2017, van Haastert *et al.* 2017, Baniukiewicz *et al.* 2018, Cheng and Mullins 2020, Lee *et al.* 2020, Ghabache *et al.* 2021, Banerjee *et al.* 2022, 2023, Pal *et al.* 2023, Tong *et al.* 2023, Chua *et al.* 2024, Gong *et al.* 2024, Lin *et al.* 2024, Deng *et al.* 2025, Iwamoto *et al.* 2025, Kuhn *et al.* 2025). Additionally, some of these tools require user registrations or were not released under an approved free/libre license (https://www.gnu.org/licenses/license-list.html) (Free Software Foundation 2025). To our knowledge, no free and open-source standalone software fully automates membrane kymograph generation while accounting for dynamically deforming cell boundaries.

Here, we present *Membrane Kymograph Generator*, a GNU GPL v3 licensed, cross-platform GUI software that automates the entire process of generating and analyzing kymographs along dynamic boundaries from multichannel time-lapse live-cell fluorescent images. The software extracts cell boundaries, tracks and aligns them across frames using a rotational offset minimization algorithm, samples fluorescence intensities along the boundary, applies LOWESS smoothing and normalization, exports outputs in various accessible formats, generates publication-quality kymographs for each channel, and provides built-in correlation analysis tools for multi-channel datasets. A Python API enables programmatic access, batch processing, and further downstream analysis. Importantly, unlike previous tools that require knowledge in MATLAB/Python/Julia for parameter tuning, *Membrane Kymograph Generator* provides an intuitive interface suitable for researchers with any level of computational expertise.

## 2 Software architecture and results

**Figure 1:**
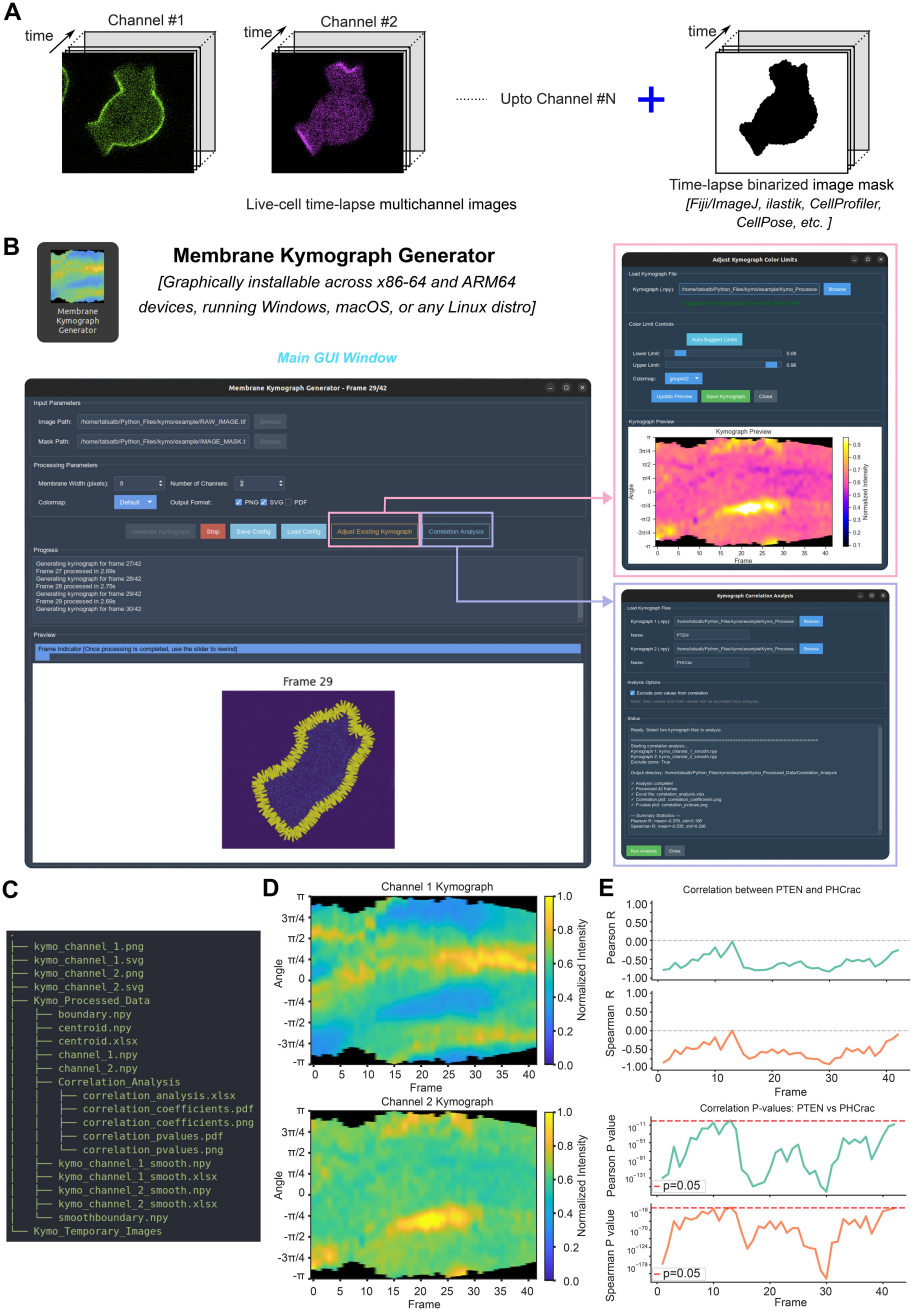
Overview of the default GUI workflow for Membrane Kymograph Generator. (A) Input files for the Membrane Kymograph Generator include a multichannel time-lapse live-cell imaging dataset and a binarized mask of the whole cell. Note that the binarized mask can be generated using various standard tools such as Fiji/ImageJ, ilastik, CellProfiler, CellPose, etc., or using custom code involving thresholding, morphological operations, or machine learning-based approaches. (B) Graphical User Interface of Membrane Kymograph Generator. The main interface is shown on the left whereas auxiliary windows are shown on the right. The main interface allows users to upload their input files, configure analysis parameters, and visualize the progress and error messages (if any) in real-time, and finally visualize the generated kymographs. The bottommost panel here shows the tracked boundaries and intensity sampling domain. The top right auxiliary window allows the users to adjust the visualization settings, such as colormap and intensity scaling. The bottom right auxiliary window facilitates the downstream correlation analysis across different kymographs. (C) List of key output files generated by the Membrane Kymograph Generator. The temporary images inside the *Kymo_Temporary_Images* folder are hidden from the tree view here. (D) Representative output kymographs generated by Membrane Kymograph Generator from two-channel live-cell images over 42 frames. Evidently, each column corresponds to a specific time point, and each row corresponds to a specific position along the cell boundary. The intensity values in the kymographs indicate the fluorescence intensity of the respective proteins at each position and time point. The colormap used is shown on the right. (E) Representative output correlation analysis plots generated by the “Kymograph correlation Analysis” module of Membrane Kymograph Generator. The top two plots show the Pearson’s and Spearman correlation coefficient (R) values between two kymographs over time, indicating the degree of correlation between the two proteins’ dynamics along the membrane boundary. The bottom two plots show respective *P* value associated with the correlation coefficients, indicating the statistical significance of the observed correlations.

*Membrane Kymograph Generator* is implemented as a Python package with additional components in bash and Inno Setup scripts. The software uses NumPy, SciPy, and Pandas for numerical operations; scikit-image, OpenCV, Shapely, and Pillow for image processing; Matplotlib and Seaborn for visualization; joblib for parallel processing; and ttkbootstrap for the graphical user interface. The modular architecture supports both GUI-based and programmatic workflows via a built-in Python API (Fig. 1).

### 2.1 Installation and updates

The software can be installed on computers with x86-64 and ARM64 architectures, running Windows, macOS, or Linux. It is primarily distributed as standalone installable packages (.exe for Windows, .dmg for macOS, .appimage for Linux). Additionally, portable binary executables and tarballs for each platform are also provided. The codebase has been tested on Windows 10/11, Ubuntu 22.04/24.04 LTS, and macOS 15. For programmatic access, the software can also be installed inside any standard Python environment using PIP from the Python Package Index (PyPI) repository (package ID: *membrane-kymograph*) or built from source. A *GitHub Actions*-based CI/CD pipeline automatically builds, tests, and deploys new versions upon tagged commits to the main branch of the GitHub repository.

### 2.2 Graphical user interface overview and input files

The GUI, built using ttkbootstrap/tkinter, provides an intuitive interface for general users (Fig. 1B–E). The main interface features organized panels where users select their multichannel time-lapse image and binarized mask files via native file browser dialogs. Users configure key parameters including membrane width for perpendicular intensity sampling (default: 8 pixels, adjustable), number of fluorescent channels (1–10), colormap (default modified Parula, plus viridis, plasma, inferno, gnuplot, and others), and output formats (PNG, SVG, PDF). A progress panel provides real-time feedback, while a preview panel displays tracked boundaries with perpendicular sampling lines overlaid on fluorescence images, with an integrated frame slider for inspecting tracking quality across the time series. The preview panel also shows the generated kymographs after processing completes. Auxiliary dialog windows allow interactive fine-tuning of intensity scaling and colormap for previously generated kymographs without reprocessing (“Adjust Existing Kymograph”), and frame-by-frame Pearson and Spearman correlation analysis between channels with significance testing (“Correlation Analysis”; Fig. 1E). Custom configuration files can be saved and loaded for consistent parameters across datasets. The software accepts standard multi-frame TIFF files (Fig. 1A), with multichannel files processed at once by specifying the channel count. All outputs are organized into structured directories (Fig. 1C), facilitating reproducibility and downstream analysis.

### 2.3 Membrane coordinate extraction

The software begins by loading the user-provided binarized mask, which can be generated using standard segmentation tools such as Fiji/ImageJ, ilastik, CellProfiler, CellPose, or custom approaches. For each frame, the cell boundary is identified using contour detection (skimage.measure.find_contours) (van der Walt *et al.* 2014), the largest enclosed area is selected, and the geometric centroid is computed. Boundary coordinates are interpolated with a piecewise cubic Hermite interpolating polynomial (PCHIP) (Fritsch and Carlson 1980), which preserves shape and prevents oscillations, and then resampled to uniform arc-length spacing to distribute boundary points evenly along the contour.

### 2.4 Inter-frame boundary alignment

Cell boundaries undergo substantial shape changes and rotations between consecutive frames due to dynamic cellular processes. The software addresses this through a rotational offset minimization algorithm that aligns boundaries across frames by exhaustively searching for the optimal angular shift. For the initial frame, the boundary is rotationally aligned to a specified angular position (default: 180 degrees) by calculating polar angles relative to the centroid and circularly shifting the boundary array. For subsequent frames, the algorithm tests all possible circular shifts relative to the previous frame’s aligned boundary. To handle varying boundary point counts, the longer boundary is subsampled by randomly excluding points until both match in size; the mean Euclidean distance between corresponding points is then computed for each shift, and the minimum-distance shift is selected. This approach ensures that homologous membrane regions are consistently tracked despite large-scale morphological changes. Aligned boundaries serve as references for the next frame, creating a temporally consistent coordinate system throughout the time series. Notably, this exhaustive search imposes no hard restriction on the inter-frame interval, although ultra-long intervals relative to the cell’s membrane dynamics may yield occasional vertical discontinuities when two membrane patches produce nearly identical alignment costs.

### 2.5 Perpendicular intensity sampling

After alignment, fluorescence intensities are sampled perpendicular to the membrane at each boundary point. For each point, a local circular arc is fit to a small neighborhood using least-squares circle fitting (circle_fit.standardLSQ; https://github.com/AlliedToasters/circle-fit) (Klear and Lauritzen 2023), providing a robust estimate of local curvature and normal direction. The perpendicular sampling direction is determined from the vector connecting the fitted circle center to the boundary point. Intensities are sampled at 0.5-pixel intervals extending both inward and outward from the boundary (default: ±8 pixels, adjustable via l_perp), using biquadratic interpolation (scipy.ndimage.map_coordinates with order = 2) (Virtanen *et al.* 2020). To reduce noise, the mean of the top 5 intensity values within each perpendicular profile is used. This process is parallelized using joblib, accelerating processing for long boundaries. The resulting intensity profiles capture both the membrane-localized signal and the immediate cortical region, enabling quantification of proteins and lipids enriched at or near the plasma membrane. These intensity profiles form the basis for subsequent kymograph generation and analysis. The fidelity of these profiles depends on the upstream mask quality: while small mask offsets are tolerated by the ±l_perp sampling window, heavily over-smoothed masks may miss fine features such as filopodia or sharp invaginations.

### 2.6 Kymograph generation and normalization

Intensity profiles from all boundary points and frames are organized into kymograph matrices (boundary position × time). A locally weighted scatterplot smoothing (LOWESS) algorithm (statsmodels.nonparametric.smoothers_lowess) (Cleveland 1979) is applied along the spatial dimension for each frame with a smoothing fraction of 0.1, filtering high-frequency variations while preserving genuine features. The kymograph is then normalized using the 2nd and 98th percentiles of the raw intensity distribution, linearly rescaled to [0, 1], and clipped, ensuring consistent visualization across channels and conditions. This normalization strategy is particularly effective for live-cell fluorescence data, where photobleaching and differences in expression levels can complicate quantitative comparisons. Normalized kymographs are saved as NumPy files (.npy) and Excel spreadsheets (.xlsx).

### 2.7 Visualization and export

The software generates publication-quality kymograph visualizations with customizable colormaps, including the default Parula colormap (adapted from MATLAB; https://www.mathworks.com) (MathWorks 2025). Intensity scaling can be adjusted interactively. Kymographs are exported as high-resolution PNG (300 DPI), SVG, and PDF, alongside raw data matrices (.npy and .xlsx). Preview images showing tracked boundaries with sampling lines are generated for verification. All outputs are organized in structured directories (Kymo_Processed_Data and Kymo_Temporary_Images).

### 2.8 Multi-channel correlation analysis

For multi-channel datasets, a built-in correlation module quantifies spatiotemporal relationships between membrane-associated proteins or biosensors. The software computes both Pearson and Spearman correlations between two kymographs on a frame-by-frame basis, with optional exclusion of zero-valued pixels to avoid background artifacts. Statistical significance is assessed via *P*value, and the software generates time series plots of correlation coefficients and *P*value with significance thresholds (Fig. 1E). All results are exported as Excel spreadsheets and publication-quality plots (PNG and PDF), facilitating investigation of co-localization dynamics and functional coupling between signaling components.

### 2.9 Python API, batch processing, and further analysis

Beyond the GUI, the software provides a Python API (membrane_kymograph) for programmatic access and batch processing. Users instantiate a KymographProcessor object and call its process() method with image/mask paths and parameters. The modular architecture allows advanced users to access intermediate processing steps, such as boundary extraction, alignment, and intensity sampling, for custom analyses. The API also supports parallel processing through Python’s multiprocessing capabilities. The *GitHub Wiki* provides comprehensive documentation with example scripts for common batch processing patterns, including handling complex parameter configurations via CSV files, interrupted workflow resumption, and intensity profiling at specific spatiotemporal locations. The exported NumPy arrays can be readily imported into scientific Python libraries for advanced analyses including feature extraction, clustering, and predictive modeling, extending the utility of the software beyond interactive single-cell analyses.

### 2.10 Dissecting the dynamics of Ras/PI3K/F-actin network in migrating cells using the software

We validated the software using microscopy images from cells across the phylogenetic tree with diverse membrane-associated proteins/lipids, in different physiological conditions. Combining GUI and API modes, >100 cells have been processed as they were migrating or making macropinosomes. As a proof of concept, here we present data from *Dictyostelium* amoeba, human neutrophils, and murine macrophages, collectively dissecting the evolutionarily conserved Ras/PI3K/F-actin network during amoeboid migration under both stochastic and receptor-driven activation. A key aspect of migration is symmetry breaking at the membrane-cortex as specific components asymmetrically localize to front-state/protrusion or back/basal-state regions (Ridley *et al.* 2003, Banerjee *et al.* 2025). These asymmetric distributions play key roles in organizing cellular polarity and regulating migration. Our kymographs and correlation analysis show that PTEN and PI(3,4,5)P3 biosensor PH${\;}_{\mathrm{Crac}}$ consistently maintain tight complementarity in migrating *Dictyostelium* cells (Fig. 1D and E; Fig. S1A). In HL-60 neutrophils, newly-polymerized F-actin biosensor Lifeact shows phases of activation and extinction during migration (Fig. S1B and C): Lifeact remained largely absent during contractions but showed strong membrane recruitment during protrusions. Ras activation biosensor Raf${\;}_{\mathrm{RBD}}$ and PH${\;}_{\mathrm{Crac}}$ show strong positive correlation with slight phase difference (Fig. S1D–F), indicating Ras positively regulates PI3K at the leading edge. We also studied receptor-driven activation using RAW 264.7 macrophages: upon global C5aR activation, PI(3,4,5)P3 biosensor PH${\;}_{\mathrm{Akt}}$ shows robust membrane recruitment (Fig. S1G–I), with temporal profiles showing sharp increase post-stimulation followed by adaptation (Fig. S1I), consistent with known dynamics of C5aR-PI3K signaling axis. Together, all these observations validate the accuracy and reliability of our software as these are consistent with previous findings in the field that delineated the roles of internal feedback loops in regulation of membrane-associated signaling network dynamics.

## 3 Discussion

We have developed *Membrane Kymograph Generator*, a free, open-source, cross-platform software that addresses a significant gap in quantitative live-cell imaging analysis by fully automating kymograph generation and downstream analysis along dynamic cell boundaries. This software removes substantial technical barriers that have historically limited membrane dynamics analysis to specialized computational researchers or forced reliance on time-consuming, manual/error-prone approaches. The modular Python architecture features both an accessible GUI for routine use and a Python API for programmatic access, accommodating users from experimentalists seeking straightforward kymograph generation to computational researchers requiring batch processing and custom analysis pipelines.

We view *Membrane Kymograph Generator* as an important addition to the broader ecosystem of bioimage analysis tools and users should choose according to their specific scientific question. Static, user-defined line kymographs remain best served by *KymographBuilder* and *Multi Kymograph* in Fiji/ImageJ. For quantifying intracellular transport in neuronal projections, *KymoAnalyzer* is a suitable option (Neumann *et al.* 2017). Specific whole-cell morphodynamics analyses such as edge velocity maps or protrusion-retraction cycles can be handled by tools like *QuimP* (Bosgraaf and Van Haastert 2010) and related platforms. Upstream cell segmentation and tracking tools, as we mentioned earlier, can complement rather than compete with our software; a typical workflow uses one of these tools to produce the binary mask that *Membrane Kymograph Generator* then uses. *Membrane Kymograph Generator* is the natural choice when the question is specifically about the spatiotemporal dynamics of membrane- or cortex-localized proteins and lipid biosensors along the entire deforming cell boundary, when reproducibility and accessibility to non-programming users are priorities, or when batch processing across many cells is required.

At present, the software works as a native desktop application; in future, a cloud-native version could enable analysis of very large datasets and collaborative sharing. The software currently processes only one cell per binary mask (the largest connected component is selected when multiple are present); future versions may support multiple labeled cells from a single mask. Additional quantitative modules, such as automated spatiotemporal feature detection, frequency analysis of membrane oscillations, and machine learning-based feature extraction, could also be integrated; currently, such analyses require custom programs using our Python API.

In summary, *Membrane Kymograph Generator* provides the cell biology and biophysics communities with a tool for automated, reproducible, and quantitative analysis of membrane dynamics from live-cell imaging data. Its combination of robust algorithms, user-friendly design, cross-platform availability, and open-source licensing makes it accessible to a broad range of researchers and a valuable addition to the ecosystem of open-source bioimage analysis tools. We anticipate that this software will facilitate new discoveries delineating novel spatiotemporal regulation of membrane-associated signaling and cytoskeletal processes across diverse cellular systems and physiological contexts.

## Supplementary Material

btag400_Supplementary_Data

## Data Availability

All data needed to evaluate the conclusions of the paper are present in the main text or in the supplementary materials or in the GitHub repo (https://github.com/tatsatb/membrane-kymograph-generator). Any additional requests for information or data will be fulfilled by the corresponding authors upon reasonable request.
