# Supplementary material for: Membrane Kymograph Generator: a cross-platform GUI software for automated generation and analysis of kymographs along dynamic cell boundaries
Source: Bioinformatics. 2026 Jun 18;42(6):btag400. doi: 10.1093/bioinformatics/btag400 (PMC13310011; doi:10.1093/bioinformatics/btag400)
Supplement: btag400_Supplementary_Data [file btag400_supplementary_data.pdf]

Membrane Kymograph Generator: A cross-platform GUI software for automated generation and analysis of kymographs along dynamic cell boundaries

Tatsat Banerjee[1,2,3,*], Bedri Abubaker-Sharif[1,4], Peter N. Devreotes[1,5,*], Pablo A. Iglesias[1,4,6,*].

[1]Department of Cell Biology and Center for Cell Dynamics, School of Medicine, Johns Hopkins University, Baltimore, MD, USA.
[2]Department of Chemical and Biomolecular Engineering, Whiting School of Engineering, Johns Hopkins University, Baltimore, MD, USA.
[3]Present address: Howard Hughes Medical Institute, Whitehead Institute for Biomedical Research, Massachusetts Institute of Technology, Cambridge, MA, USA.
[4]Department of Biomedical Engineering, Whiting School of Engineering and School of Medicine, Johns Hopkins University, Baltimore, MD, USA.
[5]Department of Biological Chemistry, School of Medicine, Johns Hopkins University, Baltimore, MD, USA.
[6]Department of Electrical and Computer Engineering, Whiting School of Engineering, Johns Hopkins University, Baltimore, MD, USA.

* Corresponding authors. Email: tatsatb@jhu.edu (to T.B.), pnd@jhmi.edu (to P.N.D.), pi@jhu.edu (to P.A.I.).

SUPPLEMENTARY INFORMATION

This file contains:

- Supplementary Figure 1.

- Links to detailed documentation.

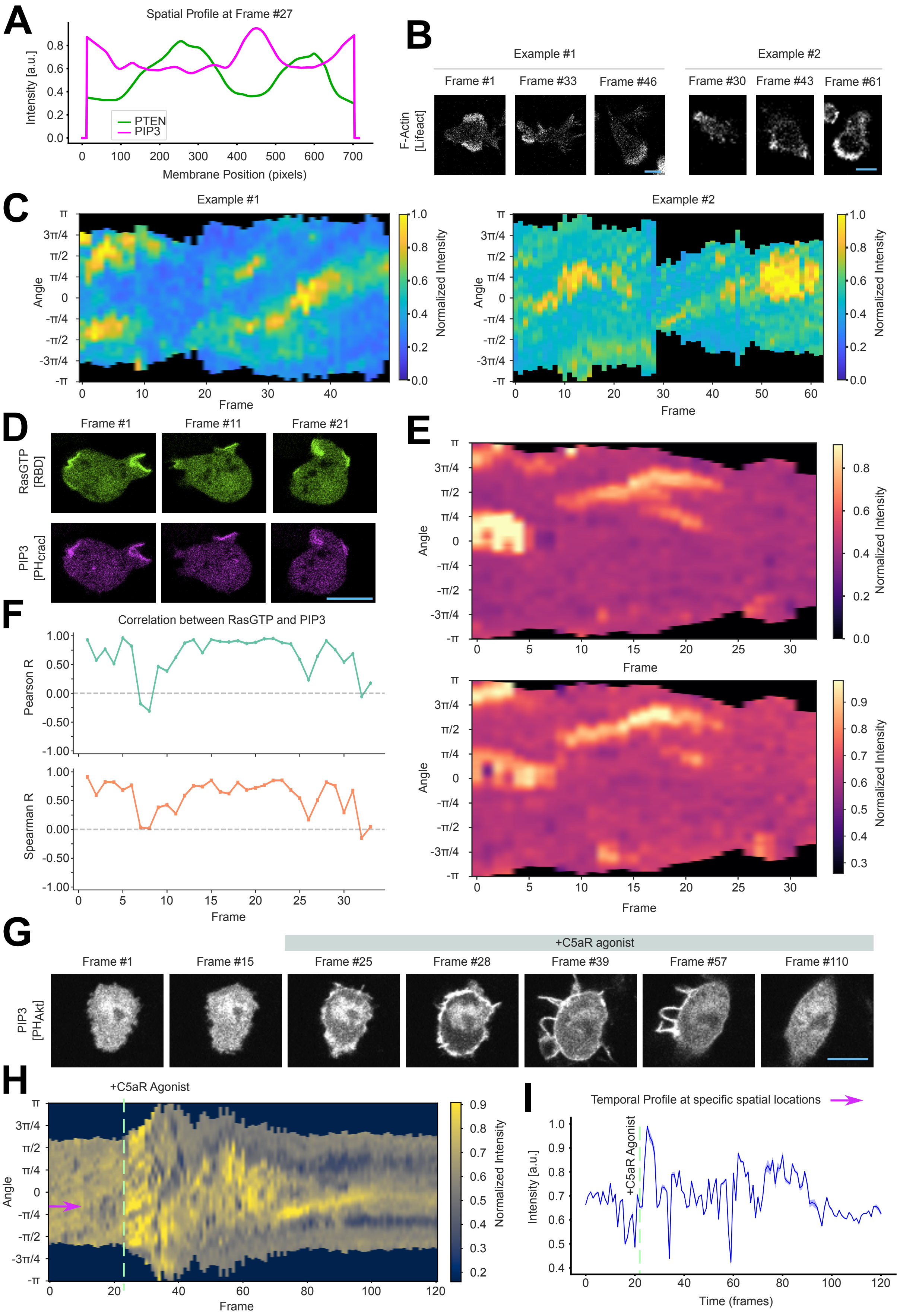
A
Spatial Profile at Frame #27
Intensity [a.u.]
Membrane Position (pixels)
PTEN
PIP3
B
Example #1
Example #2
Frame #1
Frame #33
Frame #46
Frame #30
Frame #43
Frame #61
F-Actin [Lifeact]
C
Example #1
Example #2
Angle
Frame
Normalized Intensity
D
Frame #1
Frame #11
Frame #21
RasGTP [RBD]
PIP3 [PHcrac]
E
Angle
Frame
Normalized Intensity
F
Correlation between RasGTP and PIP3
Pearson R
Spearman R
Frame
G
+C5aR agonist
Frame #1
Frame #15
Frame #25
Frame #28
Frame #39
Frame #57
Frame #110
PIP3 [PHAkt]
H
+C5aR Agonist
Angle
Frame
Normalized Intensity
I
Temporal Profile at specific spatial locations
+C5aR Agonist
Intensity [a.u.]
Time (frames)

Supplementary Figure 1 | **Membrane kymographs and downstream analyses across different cell types using Membrane Kymograph Generator.**

(A) Spatial profile of PTEN (green) and PI(3,4,5)P3 biosensors (magenta) along the membrane boundary of a migrating *Dictyostelium* cell, at a particular frame. The kymographs generated in Figure 1D of the main manuscript were used as the data. A similar example has been documented in Python API section of the wiki.

(B, C) Representative live-cell time-lapse fluorescence images (B) and membrane kymographs (C) of HL-60 human neutrophil cells expressing newly-polymerized F-actin biosensor Lifeact, during random migration. The images were acquired at 7 sec/frame rate. In both examples, "Default" colormaps were used. Here and everywhere else, scale bars indicate 10 μm (unless otherwise specified).

(D-F) Representative live-cell time-lapse fluorescence images (D), membrane kymographs (E), and time-series correlation analysis plots. The outputs in (D) and (E) were generated by the *"Membrane Kymograph Generator"*, from cells shown in (D). Here, *Dictyostelium* cells were co-expressing biosensors for Ras activation (RBD_{Raf1}-GFP) and PI(3,4,5)P3 (PH_{Crac}-mCherry) and the images were acquired at 7 sec/frame rate. The "magma" colormap was used in the membrane kymographs; note that, intensities were adjusted here after generation of kymographs for better representation (using "Adjust Existing Kymograph" module). Pearson R and Spearman R values were computed using "Correlation Analysis" module.

(G-I) Representative live-cell time-lapse fluorescence images (G), membrane kymographs (H), and temporal profile plots (I). The outputs in (H) were generated by the *"Membrane Kymograph Generator"*, from cells shown in (G) and "cividis" colormap was used in the kymograph (using "Adjust Existing Kymograph" module). Here, RAW 264.7 murine macrophage cells were expressing PI(3,4,5)P3 biosensor (PH_{Akt}-mCherry) and C5aR receptors were globally activated in the middle of the imaging experiment by adding a saturating dose of C5aR agonists (as shown with vertical lines), leading to robust activation of PI3K in the membrane. The images were acquired at 12 sec/frame rate. The temporal profiles of mean intensities (I) were computed from the kymographs in (H), by loading the raw kymograph data and taking average of 175-185 spatial positions along the membrane boundary at each time point. The selected spatial positions at the beginning of time are marked with magenta arrow (H and I). The data in (I) is mean ± s.e.m. from all those spatial positions. The (G) and (H) panels show that PI(3,4,5)P3 levels rapidly increase at the membrane following C5aR activation, peaking right after post-stimulation before gradually declining towards baseline levels over the next several frames.

Links to detailed documentation:

For comprehensive guidance on installation, usage, GUI features, the Python API, and troubleshooting, users are encouraged to consult the GitHub Wiki of *Membrane Kymograph Generator*. Key pages include:

- Quick Start Tutorial.
- Installation Guide.
- GUI Overview.
- Input and Output Files.
- Configuration Files.
- Advanced Usage: Python API.

For bug reports, feature requests, and community discussions, please use the GitHub Issues and Discussions pages of the repository.
